# Empowerment evaluation of a Swedish gender equity plan

**DOI:** 10.3402/gha.v7.23710

**Published:** 2014-07-02

**Authors:** Georgios Gavriilidis, Nivetha Natarajan Gavriilidou, Erika Pettersson, Eva Renhammar, Anna Balkfors, Per-Olof Östergren

**Affiliations:** 1 Division of Social Medicine and Global Health, Social Medicine and Global Health, Lund University (LU), Malmö, Sweden; 2 City of Malmö, Commission of a Socially Sustainable Malmö, Malmö, Sweden

**Keywords:** policy, empowerment, evaluation, gender equity, policy empowerment index

## Abstract

**Background:**

Empowerment is essential for gender equity and health. The city of Malmö, Sweden, has formulated a development plan for gender equity integration (GEIDP). A ‘Policy Empowerment Index’ (PEI) was previously developed to assess the empowerment potential of policies.

**Objectives:**

To pilot-evaluate the GEIDP’s potential for empowerment and to test the PEI for future policy evaluations.

**Design:**

The GEIDP was analyzed and scored according to electronically retrieved evidence on constituent opinion, participation, capacity development, evaluation–adaptation, and impact.

**Results:**

The plan’s PEI score was 64% (CI: 48–78) and was classified as ‘enabling’, ranging between ‘enabling’ and ‘supportive’. The plan’s strengths were: 1) constituent knowledge and concern; 2) peripheral implementation; 3) protection of vulnerable groups; and 4) evaluation/adaptation procedures. It scored average on: 1) policy agenda setting; 2) planning; 3) provisions for education; 4) network formation; 5) resource mobilization. The weakest point was regarding promotion of employment and entrepreneurship.

**Conclusions:**

The PEI evaluation highlighted the plan’s potential of constituency empowerment and proposed how it could be augmented.

Gender equity and women’s empowerment are social determinants of health (1). Power imbalances result in unequal health access and outcomes, knowledge, skills and employment, living conditions, and opportunities (2). Public policies have major impacts on empowerment (3–7).

Despite the European Union’s (EU) gender equity recommendations, women’s empowerment and inequities in individual, social, economic, and professional conditions remain a challenge in Europe (8, 9). Sweden has the tenth highest human development index, the highest gender equality and the lowest income inequality worldwide (10–12). Gender equity is considered fundamental in Swedish internal policy development (13), and the country has signed the EU plan for gender equity’s integration (9, 14). However, differences exist between socially and economically vulnerable groups and women and men on living conditions, education and health, power and influence in society as well as on employment and unpaid work distribution (15). In addition, Malmö has the most ethnically diverse population among Sweden’s big cities (16, 17). In 2011, the Malmö city office (henceforth, City) developed a gender equity integration development plan (GEIDP), in accordance with EU and national guidance. According to the plan, latest by the year 2013, all of the city agencies and services should be working toward equal distribution of services, resources, power and influence irrespective of gender, orientation, and background, and, as the city’s biggest employer, the City should provide equal employment opportunities, conditions, and salaries, and move toward a balanced gender distribution at all work categories (14).

An evaluation index named ‘Policy Empowerment Index’ (PEI) has been developed by Gavriilidis and Östergren (18), aiming to increase understanding on how policy planning can affect constituent empowerment. It aspires to do so by assessing the policy elements that affect empowerment through evidence reviews, stakeholder, and constituent feedback.

Evidence-based evaluations are necessary for more gender-sensitive and empowering policy planning. A few comparative evaluations have been attempted using ad hoc criteria and policy document content analyses (19–21); however, generally accepted criteria for such evaluations are lacking.

This study aimed to pilot-evaluate the Malmö GEIDP from the perspective of comprehensive empowerment of its constituents, in order to contribute with evidence-based feedback to the policy discussions and to evaluate and develop the PEI for more comprehensive and systematic future policy evaluations.

## Methods

The PEI was previously developed to assess policy elements that have a potential impact on empowerment. It applies logical and discussed scores (from 0=minimum to 5=maximum) of empowerment potential (18) and confidence intervals (0 to 5). Due to lack of evidence on empowering factors’ impacts, the scores are not evidence-weighted. To make the results more visible, the PEI evaluators attach scores to logical statements. For example, <10% describes ‘a small minority’, 10–50% ‘a significant minority’, 50–75% ‘a majority’, 75–90% ‘a big majority’, and 90–100% ‘a vast majority/almost all’, as previously described (18).

A pilot evaluation of the plan was performed after a few adaptations: Questions (Q) 10 and 11 (on policy evaluation and adaptation) were fused, and Q12 was removed (investigation of related policies) for simplicity (see Appendix 1). The remaining 10 questions interrogated the policy plan on the following issues: 1) participation; 2) capacity building; 3) evaluation/adaptability. The index questions cover these policy empowerment elements as follows:

- Constituent concern with the policy issue and participation in agenda setting (Q1, 2), policy planning (Q3), and implementation (Q4).
- Building constituent capacities and opportunities through education/training (Q5), employment/entrepreneurship (Q6), network formation (Q7), addressing power inequalities (Q8), and resource mobilization (Q9).
- Modes of policy evaluation (Q10).

The PEI questions the evaluation of the electronically retrieved evidence addressing them; the PEI standards and scores are presented in Appendix 1. Each question’s argued scores and CI of the GEIDP are averaged to generate a total score and CI, which are presented as proportions of the maximum 50 for intuitiveness. A summary and discussion of the evaluation and the final result is presented in ‘Results’ and in Table 1.

**Table 1:** Summary of PEI evaluation, City of Malmö, development plan for integration of gender equity

Q1. How many political constituents (residents in any way affected by the plan) are informed and concerned with the addressed problem?	Score: 3.5 (3–5)
Q2. How was the political agenda set? Did the plan in question start from a discourse in the community and local grassroots movement advocacy, or by professional experts and politicians at the City level and above (or both in interaction)?	Score: 3 (2–4)
Q3. How was the policy planned? Did peripheral agencies and interest groups contribute significantly to the planning?	Score: 2.5 (2–3)
Q4. What percentage of the development plan actions was delegated peripherally for implementation? Will the plan be implemented mainly by the central or peripheral City authorities?	Score: 5 (4–5)
Q5. Does the policy plan call for education/training of the constituents?	Score: 2 (1–3)
Q6. Is peripheral employment and entrepreneurship being strengthened? Will the plan create jobs or business opportunities for women or men?	Score: 1 (1–2)
Q7. Does the plan promote constituent participation in horizontal and vertical networks? Does the plan create links between the community members/citizens/residents and between them and the authorities of the city?	Score: 2 (1–3)
Q8. Are hard to reach, vulnerable or disadvantaged populations being considered and affirmatively protected and empowered (including vulnerable gender and age groups, socially/physically/economically disadvantaged individuals, groups and communities)?	Score: 5 (4–5)
Q9. Does the policy provide for or will there most likely be adequate financial, human and other resources?	Score: 3 (2–4)
Q10. How will the policy plan be evaluated and adapted?	Score: 5 (4–5)
**Total: 32 (23–39)**	**Score: 64%**
**Policy classification: enabling (supportive–enabling)**	**CI (46–78)**

According to the PEI, a policy can be classified as ‘Dictative’ (score 0–20%), a top–down policy that disregards constituent empowerment, or ‘Empowering’ (81–100%), an optimally focused, planned, and evaluated policy with empowerment in central focus, ‘Directive’ (21–40%), ‘Supportive’ (41–60%) or ‘Enabling’ (61–80%) being the intermediate policy categories as their names imply [see Appendix 2 for the full definitions of these policy categories, as in (18)]. These policy categories are ideal, theoretical models. Real policies may contain elements from more than one policy type, which average on the category indicated by the PEI score.

We conducted evidence and public debate/opinion searches to address each PEI question on constituent opinion and participation in plan design, implementation and evaluation/adaptation, and capacity development through education, employment, networks, and affirmative support. The following search queries were performed on Google, Google Scholar, Web of Science (WoS), and PubMed in English and Swedish using the following search queries: [‘gender equity’ AND ‘study’], [‘gender equity’ AND ‘study’ AND ‘Malmö’], [‘gender1 equity’ AND ‘study’ AND ‘women’], [‘gender equity’ AND ‘study’ AND ‘immigrant’], [[‘survey’ AND [‘gender’ OR ‘women’] AND [‘equity’ OR ‘equality’] AND [‘Sweden’ OR ‘Malmö’]], [‘survey’ AND ‘gender’ AND ‘immigrant’], [[‘gender’ AND [‘equity’ OR ‘equality’] AND Sweden]]. Only the first 100 search results from Google were examined (total 600). Relevant data such as education and employment statistics and official documents were retrieved from ‘Statistics Sweden’ (SCB), the ‘City of Malmö’ webpage, and through contact with city employees. The retrieved documents and statistics were selected according to relevance to each of the search questions and to Malmö, and broader inferences to Sweden were included in the absence of local ones.

The PEI evaluations can optimally be performed by many evaluators and with stakeholder feedback through surveys, interviews, and discussions (18); however, due to practical constraints it was only based on a literature review performed by two of the co-authors (GG and NN). The evaluation report was submitted to the ‘Commission for a Socially Sustainable Malmö’ and was included as an element in its final proposal (22, 23).

## Results

A total of 778 documents were retrieved and examined for relevance on Google and Google Scholar (600), PubMed (54), and WoS (124). After examination for relevance and repetition, 70 documents from Google, 30 from PubMed, and 61 from WoS (total 161) were used for the evaluation.

The complete evaluation and bibliography are presented in Appendix 1. The questions and evaluation scores are summarized in Table 1. Below we summarize the GEIDP evaluation findings:

### Constituent concern and participation

Q1. The GEIDP addresses the persistence of gender inequity in Malmö, for example, the inequities in employment, living conditions, education and health, power and influence in society, wages, expectations, unpaid work distribution and parental leave, sexual harassment, and gender-based violence. The evaluation suggests that between 50 and 90% of Malmö residents are currently concerned with gender inequality at home and work, and with associated issues such as gender-based violence.

Q2. Gender equity is of widespread concern in Sweden and internationally, and the agenda for this policy appears to have been set beyond the city level, although non-expert local constituents (e.g. party members) have raised the issue in Malmö. The EU and State roles in driving the issue toward the City Council and Board seem to dwarf the local community contributions.

Q3. This is a centrally conceived policy plan with significant but not determining peripheral feedback by local authorities (such as local councils), agencies (such as the educational, technical and cultural agencies) and interest groups (such as the teachers’ union), some of which had developed their own plans for gender equity. Q2 and Q3 could profit from further discussion and input from the policy-planners.

Q4. The plan is to a very large extent delegated to the peripheral authorities, agencies, departments and businesses for implementation. A number of city departments and agencies have already designed their adapted plans. However, the overall control, supervision and support are left to the central authority.

### Building constituent capacities and opportunities

Q5. The plan relies significantly and invests on education on gender equity issues among the City employees at all levels but mainly for coordinators and mention is made for education as a means of more equitable social integration. Gender education is targeted toward students of all levels, from primary school to university and leaders. However, no specific programs for targeted training in areas where women or men are lagging behind are planned although some especially vulnerable groups are supported for job seeking by publicly supported programs.

Q6. There is some direct creation of employment through this plan, mainly in its implementation in education on gender, gender sensitive budgeting and communication and in application of gender disaggregated statistics as well as, indirectly, through developmental benefits, private sector attitude change and fairer parental leave distribution. However, there is no explicit provision for extra jobs; therefore the impact on jobs seems unlikely to exceed 1% of the city’s employable residents (i.e. aged 16–64).

Q7. The plan calls directly for the responsibility of the City to support gender equity contact persons of peripheral administrations and companies through an existing website (malmo.se/jamstalldhet) and the city intranet (KomIn). Indirectly, it aims to place new emphasis on the issue, by spreading knowledge and serving as a model for wider equity in the society. The discussions and seminars are likely to improve the communication between the many groups concerned with gender equity, institutions, agencies and businesses active in Malmö and between those and the City. Given the large number of existing groups, it is unlikely that many new groups and networks will be formed because of the plan. Therefore, the proportion of constituents that the plan will indirectly motivate to participate in these links is unlikely to exceed 10% of the resident population.

Q8. Despite living in one of the world’s most equitable societies, many women in Sweden can still be considered disadvantaged and vulnerable. Seventy two percent of all leaders and 82% of board members of publicly listed companies are men. Only 16% of professors are women and a woman’s average salary is 8% lower than a man’s. The aggregate total income of women is 70% of men’s while they contribute threw times more in unpaid services. One in five women feels that she is being treated unequal at work and one in four is unhappy with gender equity in this country. There are over 25,000 crime reports, mostly by intimate partners, and around 6,000 rapes against women every year. Malmö is 37th among 290 municipalities on the SCB’s gender equity index (Jämindex), last among the major cities. Forty percent of its population has an immigrant background and 30% were actually born abroad. Immigrant women often face even more challenges toward employment and empowerment. Men are also disadvantaged in regards to parental leaves.

The plan calls directly for improvement in the both genders situation among the City’s employees first, and through the handling of public matters to all the gender-disadvantaged residents of Malmö, clearly more than 10% of the population.

Q9. Central (City Council) funding and resources (mainly human) are called for, which are most likely to be sufficient since significant funds have already been budgeted and allocated and the City currently has sufficient financial assets and budget commitments were made. Given the local and national academic environment’s focus on gender knowledge and human resources are also most likely to be available.

### Modes of policy evaluation

Q10. The plan calls for annual, gender disaggregated statistics of measurable targets and consequence reports from the city boards and administrations to be continuously created, and an annual revision update report and analysis will be presented by the city board. The City will be responsible for the overall follow-up of gender equity integration′s progress by the city administrations and also provide support and coordination (24), p. 15). In addition to the statistics the plan explicitly asks for qualitative equity analyses, in order to sample the ‘residents and users experiences through different kinds of surveys’ (24, p. 24). The City Council must approve changes in the plan. However, as adequate authority is delegated peripherally for implementation, several city departments and administrations have already formulated and adapted the plan to their situations so this is also likely to happen after the evaluations.

### Evaluation conclusions

After applying the index scores to these questions (see Table 1 and Appendix 1), a total score of 64% (46–78%) best describes the policy plan as ‘Supportive’ of empowerment and ranging between the ‘Supportive’ and ‘Enabling’ PEI policy categories (see Appendices 1 and 2).

The policy plan’s strong points according to the index are: 1) the wide constituent knowledge and concern with gender inequality (Q1); 2) the peripheral implementation by the city departments, administrations, agencies and companies (Q4); 3) the protection conferred to women and men against gender discrimination by the City as employer, and the push for support by the City agencies for a more equitable society and empowerment through employment, education and fairer distribution of resources and agency (Q8); and iv) the comprehensive evaluation and adaptation procedures in the city (Q10).

The plan scored average on the following points:

1. A centrally set policy agenda (Q2). Although initiated by grassroots movements, the gender equity agenda in Sweden is nowadays pushed forward by international and national agencies, outside the constituent communities. One central assumption of the PEI is that policies are more empowering when their constituents own them from their conception. Obviously the plan is part of a wider policy framework, which has sprung from population movements in the mid-1900s. However, the PEI here estimates its own empowering potential, and not that of the overall gender equity policy.
2. The planning was done mainly by City-office experts, with no game-changing peripheral and lay contributions (Q3). Active, wide and equitable constituent participation in policy planning may be effort- and time-consuming but has a potential to empower by delegating agency and influence, and through better responding to current and consensual community needs.
3. The plan’s provisions for education are focused on gender issues (Q5). Additional capacity development could stem from more explicit support to skill development to overcome proficiency inequalities.
4. Emphasis is placed on communication, but new network formation is limited (Q7). The existing networks could always benefit from more vivid and wide participation. Maximization of contacts and engagement of different views can empower the community toward coming up with innovative solutions and faster norms’ change, benefiting from Malmö′s cultural diversity.
5. Resources may be adequate, but more local, creative resource mobilization, e.g. advice for promotion of volunteer work, or even a community ‘inequality tax’ could generate local funds for equity programs, promoting local ‘ownership’ of the plan’s components (Q9).

According to this evaluation, an issue where the plan can be improved from empowerment’s perspective is on promotion of specific and targeted employment and entrepreneurship (Q6). The City has the capacity to promote equitable employment not only as an employer but also through its regulations, services and programs. It can be argued that unemployment levels in the city are relatively low. However, disadvantaged groups certainly exist, such as within ethnic, age groups, or with specific professional orientations, penal and addiction histories. Part time or temporary work is also problematic for sustainable career development, economic independence and empowerment.

## Discussion

We conducted a pilot evaluation of a Swedish gender equity plan using an empowerment index, based on diverse evidence sources, ranging from official documents, statistics and peer reviewed articles to published and electronic news and debates. Our aim was to provide feedback to the policymakers on the GEIDP as well as to test and develop the PEI for future policy evaluations for empowerment.

There is a clear need for a richer policy discourse and feedback on policy design (25). Public policies are most commonly evaluated after their implementation, whenever this is possible. This is easier for some policy types, such as monetary and health policies, where the impacts can often be measured. Few others have focused on various models of policy document content analysis according to predefined criteria, which apply best to gender equity policies (19–21). Thus, they have been able to compare gender equity provisions and gender mainstreaming among health policies. Such approaches are obviously valuable for policy discourse and development; however, more systematic and generally accepted and applicable, synoptic tools such as indexes should be developed to turn the art into science. To date, no evaluations of constituent empowerment by policies have been found other than our previous evaluation of the South African traditional medicine policy plan (18). That policy was found to be less empowering that the GEIDP [PEI score 42% (27–57)], mainly due to minor peripheral participation in the planning and implementation; however, comparisons among policies with such different scopes and contents using the PEI, although of interest from an overall empowerment perspective, are in need of further validation and standardization of the index and its methodology. We argue that empowerment of the constituents is important for democratic policy planning and that planning stage evaluations using standardized tools can contribute toward better-designed policies. The PEI is an effort in that direction.

This evaluation has significant limitations. Concessions were made in regards to the number of evaluators and the sampling of stakeholder responses due to time and staff availability constraints. The PEI evaluation can optimally be performed by three or more evaluators including members of the constituency, and combined with more extensive surveys.

However, an extensive review of both official and lay documents on the world’s broadest discussion forum, the World Wide Web, strengthens the validity of the findings. The use of both official and unofficial sources in the evaluation enriches the discourse about the plan, including sources that may be ignored by policymakers. We also communicated with a member of the policy-making team who responded to six out of the 10 PEI questions, suggesting a score of 80%, where our score for those questions was 66%. Similar feedback from several policy stakeholders could further validate our findings.

The PEI may be inadequate for comprehensive feedback to policy planners. However, it can be combined with existing and further developed socioeconomic, political, and health indicators such as the Gini income inequality index, OECD indicators (26), the gender equity index (Jämindex) (27), the Gender-related Development Index (GDI), the Gender Empowerment Measure (GEM), the Gender Gap Index (GGI), the Gender Status Index (GSI), The African Women’s Progress Scoreboard (AWPS), and the UNDP Gender Mainstreaming Scorecard (28, 29). More comprehensive indexes and indicators, for example, also measuring well-being and sustainability, are needed for a more holistic understanding of social welfare and progress (30).

Also, a single policy plan evaluation may be inadequate. Complementary plans and strategies may increase the overall policy’s empowerment potential. Therefore, a multi-sectorial policy ‘cluster’ evaluation is proposed as the next step.

## Conclusions

An empowerment evaluation of the Malmo City’s policy plan to increase gender equity in all aspects of city life showed that the plan has a strong potential to empower its constituency, being, according to the PEI terminology, ‘supportive’ and potentially even ‘enabling’ of empowerment’ with a PEI score of 64% (46–78%) (see Appendix 1). The plan’s main strong point according to this evaluation lies in the protection against gender discrimination in employment, education and distribution of resources and agency. Proposals that emerge from the analysis are toward proactive job creation for vulnerable groups and more delegated policy conception and planning.

Comprehensive policy evaluations can contribute toward a more participatory, community enriching and effective policy planning, facilitating the achievement of developmental and public health goals. The PEI can be a part of such evaluations.

## Appendix 1 Results of the Policy Empowerment Index Evaluation of the Malmö city’s Gender Equity Integration Development Plan

The full report can be found at the Malmö city’s web site: http://www.malmo.se/download/18.31ab534713cd4aa921379ea/Prooposal+inc+evalutation.pdf

Q1. How many policy-affected constituents are informed and concerned with the problem being addressed?

  0) No one

  1) <10%,

  2) 10–50%,

  3) 50–75%,

  4) 75–90%,

  5) 90–100%, of constituents

According to the evidence search:

The problem that the GEIDP addresses is the persistence of gender inequity in Malmö, for example, the inequities in employment, living conditions, education and health, power and influence in society, wages, expectations, unpaid work distribution and parental leave, sexual harassment and gender-based violence.

According to recent surveys, over 75% of Swedish men and women support a balanced distribution of unpaid work and salaries, and 86% of employees believe that gender salary differences are wide (18, 32), although the populace does not prioritize gender equity over other issues (33–35). Sweden is one of the most gender equal societies (36–38), despite differences in salaries and work positions (24, 39–45), unpaid homecare and parental leave (46–57), and in healthcare (58, 59). Women are concerned about unequal opportunities and one in five women feels gender-discriminated at work (60–62). Around 60% of both men and women want a fairer distribution of parental leave (63).

Many Malmö residents are concerned about gender equity, and gender issues feature in basic and higher education curricula (64–66); however, no local surveys were found. Malmö has a higher percentage of residents with foreign background than the rest of Sweden: 30% born abroad, 41% with foreign background (20). Immigrants may have different views from natives on gender equity, but, according to global surveys, a large proportion of them are expected not only to know and be concerned but also to agree that men and women should have equal rights (60–97%), although fewer people globally believe that there is more to do about gender equity (from 35% in Egypt to 81% in France) (67, 68).

The GEIDP addresses the lack of gender equity in Malmö, beginning with safeguarding equal opportunities, conditions and rewards by the city as employer, and through the city departments′, administrations′ and companies′ services and distribution of resources (17).

In the absence of recent local statistics on Malmö resident views on gender equity, the above evidence suggests that between 50 and 90% of Malmö residents are currently concerned with gender inequality at home and work, and with associated issues such as gender-based violence, which the GEIDP aims to address. A local sampling of opinion, such as through the PSA survey, can be useful to narrow the confidence interval. *PEI Score: 3.5 (range 3–5)*

Q2. How was the political agenda set? Did the plan in question start from a discourse in the community and local grassroots movement advocacy, or by professional experts and politicians at the City level and above (or both in interaction)?

By:

  0) Some non-legitimate leader/authority, non-transparently

  1) experts, centrally

  2) experts, multi-level or peripherally

  3) mixed (experts and lay stakeholders), centrally

  4) mixed, multi-level

  5) mixed, peripherally

According to the evidence search:

Gender Equity was added to Swedish political agenda after the economic transformation and the global feminist movement (1960s), and gained political support in the 1970s (69, 70).

The current plan is a response to international guidance (71) and more specifically an EU proposal signed by national representatives (12, 72). Although many local, national and international groups with local representation and political party branches have gender equity high in their agenda (37, 71, 73–84), a relative delay in implementation in Malmö may be the result of satisfaction with the current situation and maybe even some controversy on gender equity’s social and health effects (34, 35, 63, 85–92). An interesting report suggests that evolution of family gender norms has lagged behind policy makers’ ideas (63). On the contrary, significant evidence was found for a national and international agenda for gender equity, supported by human rights, macroeconomic and macro-demographic priorities (91, 93–104).

The gathered evidence therefore suggests that gender equity is of widespread concern in Sweden and internationally (see also Q1) and thus the agenda for this policy appears to have been set beyond the city level, although non-expert local constituents (e.g. party members) have raised the issue in Malmö. The EU and State roles in driving the issue toward the City Council and Board seem to dwarf the local community contributions. However, this conclusion can be contested by survey input and further discussion with the policy stakeholders. *PEI Score: 3 (2–4)*

Q3. How was the policy planned? Did peripheral agencies and interest groups contribute significantly to the planning?

The plan/policy was drafted by:

  0) Some non-legitimate leader/authority, non-transparently

  1) experts, centrally

  2) experts, multi-level or peripherally

  3) mixed (experts and lay stakeholders), centrally

  4) mixed, multi-level

  5) mixed, peripherally

According to the evidence search:

The planning was done at the City of Malmö (105) after a stakeholder opinion evaluation and significant contributions from peripheral agencies and stakeholders, such as the community councils, the educational, technical and cultural agencies, the local house construction company (MKB) and unions (such as the teachers’) among others, through meetings and written responses to a plan draft (106).^1^

However, the planners were city employees with experience in gender issues, thus considered experts, without lay community stakeholders among them. The peripheral feedback was significant but not game-changing for the plan’s final formulation (25, 107). The plan firmly complies with the pre-existing city plan, the EU charter and the Swedish government and agencies’ guidance (12, 72, 107–111). No evidence of significant Malmö or other residents’ or agencies’ participation was found in those documents.

In addition, peripheral city departments appear to have followed the city in their planning instead of contributing their own plan proposals to the final plan formulation (112–118), although one department had already formulated a gender equity plan based on the previous guidance (113) and another had significant relevant experience (114). This issue, together with Q2 can profit from further discussion and input from policy-planners. *PEI Score: 2.5 (1–3)*

Q4. What percentage of the development plan actions was delegated peripherally for implementation? Will the plan be implemented mainly by the central or peripheral City authorities?

  0) No delegation

  1) <10%

  2) 10–50%

  3) 50–75%

  4) 75–90%

  5) 90–100%

According to the evidence search:

The plan is to a very large extent delegated to the peripheral authorities, agencies, departments and businesses for implementation (17, pp. 8, 9, 13, 14, 15), especially in regard to the city services. Only a few of its measures will be implemented at the central city level, and those mainly concern the City working environment (17, p. 8).

The City is employing less than 10% of Malmö’s active workforce, total workforce or population (23, 119–122). This proportion can be used as an indicator of the magnitude of central vs. peripheral implementation needed for the measures required by the City as an employer, e.g. education–training, recruitment and overall adaptation of the working environment.

However, overall control, supervision and support remain with the central authority, the City Board and Council (17, pp. 10, 14). The municipal boards, agencies and companies will be equipped with one or more contact persons coordinating implementation and follow-up of the plan (17, pp. 13, 14) while leaders at all levels are held responsible for the implementation (17, p. 15).

A number of city departments and agencies have already designed their adapted plans (112–114, 116, 117, 119, 123). *PEI Score: 5 (4–5)*

Q5. Does the policy plan call for education/training of the constituents?

  0) <1%

  1) 1–10%

  2) 10–50%

  3) 50–75%

  4) 75–90%

  5) >90%

According to the evidence search:

The plan relies significantly on education on gender equity issues among the City employees, at all levels but mainly coordinators and leaders (17, pp. 7, 10–12, 17, 25) and mention is made for education as a means for more equitable social integration (17, p. 19).

Commitment for advancement of gender equity can be found in the last City budget (124, Q9). The City department of education has already formulated its equity plan, with a significant number of activities for City employees, leaders and support to schools by gender educators, and more such personnel with experience in gender issues, gender equitable budget work and communication, is sought by public announcements (123, 125). In 2011, 9,000 employees were trained through role-plays at work, and 141 people were trained as play leaders ((122, 123) p. 13) on diversity issues. The total number of employees on December 31st 2011 was 20.521, increased by 545 from the previous year and the City had an annual employee turnover of 1560 (122, pp. 8, 10). If similar numbers are maintained, a little over 40,000 city employees may be employed and, according to the plan’s objectives receive some kind of training for gender equity over the plan’s implementation decade, not counting potential interns (900 in 2011) and day-workers. This accounts for approximately 18% of residents aged 6–64 (see footnote 1) (23, 126).

Regarding the plan’s provisions for the City as a service provider, evidence was found that gender education targeted toward students of all levels, from primary school to university (127–130), city employees (122, 130, 131) and leaders (125, 131), is already advancing. Clearly education on gender issues is needed to address norms that hinder gender equity at home and work (55, 132–134) and to protect women from violence and harassment (134). Even in public educational institutions, study materials (133), training programs (135, 136), and educators’ knowledge and attitudes can improve (126). The city′s population enrolled in educational programs in 2010–11 amounted to 27,328 aged 6–15 and 34,911 aged 16–64, totaling 62,239, or 27.3% of total residents aged 6–64 (see footnote 1) (23, 126, 136–139).

The question arises if more education and training can increase population empowerment in Malmö. There evidently exists a high degree of gender equity regarding both on-going education and education levels in Malmö and overall in Sweden (126, 136, 139), but significant differences exist at different areas of training, with higher proportions of men in technical positions and lower in care trainings, and a lower proportion of women in research (139). Women are also lagging behind in top job positions and entrepreneurship (39, 40, 139) and some ethnic groups of women have very low levels of official employment (24). It is thus obvious that some more targeted training (such as leadership management and entrepreneurship courses for women) and an overall correction of these differences could widen the career opportunities for significant additional population numbers and thus empower the Malmö community. However, no specific programs for targeted training in areas where women or men are lagging behind are planned (17, 123), although some especially vulnerable groups are supported for job seeking by publicly supported programs (140). Only around 17.4% of residents aged 16–64 are receiving education at the moment (23, 126, 136–139).

Thus, mainly employees and current students are eligible to receive some extra education by the plan before 2020, which accounts for around 46% of the total that is eligible to study resident population (see footnote 1) (2011). *PEI Score: 2 (1–3)*

Q6. Are peripheral employment and entrepreneurship being strengthened? Will the plan create jobs or business opportunities for women or men?’

  0) No

  1) employment/entrepreneurship for <1%

  2) employment for 1–10%

  3) employment for >10%

  4) some entrepreneurship and self-employment for 1–10%

  5) broad entrepreneurship and self-employment for >10%

According to the evidence search:

There is some direct creation of employment through this plan, mainly in its implementation.

The plan aims to eliminate gender discrimination by raising awareness on gender issues among employees and leaders, controlling unequal recruitment and remuneration, and promoting equal employment conditions and opportunities (17, p. 8) as well as guaranteeing equitable activities, services, treatment, exercise of public authority, distribution of resources, power and influence (17, p. 7). Leaders and employees will be educated on gender, gender sensitive budgeting and communication and gender disaggregated statistics and reporting will be implemented (17, p. 8–11). Educators with experience in gender issues will thus be needed and relevant provisions have already been made (123, 125). Given the large number of employees and occupations (124, p. 2), these tasks will probably require a significant amount of extra work-time.

Extra funds are earmarked for gender equity in the 2012 budget (23, pp. 26, 48). All administrations and municipal companies will assign at least one contact person responsible for the equity integrations coordination (17, p. 13). There are 10 peripheral administrations and 14 municipal companies, so this requirement could create a few new jobs. However, no explicit provision for extra jobs was found in the current budget and revision of personnel (23, 124). In any case, new educators and coordinators together will not account for more than 0.5% of the eligible to work resident population of 201,123 aged 16–64 (see footnote 1) (137, 138).

Additional employment and entrepreneurship may be created by facilitating access to ‘work, housing, culture, information–communication as well as social and medical help’ (17). Although to a large extent these services are already in place, more targeted activities are well needed (131, 140, 141) and some programs targeting young or marginalized women have already been put in place (39, 142). However, in the absence of an explicit call for job creation, the impact of such programs is expected to be proportionally low.

The plan is also unlikely to create new jobs through its goal for an equitable balance of recruitment and employment. Among the city’s 20,521 non-day-worker employees, 77.2% were women and 22.8% were men, with women also occupying more leadership positions (124. pp. 12). Sweden is now one of the few European countries with increasing gender desegregation in its labor, though a decade ago was regarded a high-employment and high-gender-segregated country in EU (143, p. 18). However, the European labor market analysis of gender segregation reports that there is a gender polarization of jobs where women, although with a higher employment rate, are more associated with ‘feminized’ jobs relating to teaching, care giving, etc., while men are involved in computing and technical jobs (143, p. 8). This could contribute to narrow skill development, labor supply and resultant biased employment opportunities (143, p. 43). This gender imbalance in favor of women implicitly serves to balance inequalities in favor of men in the private sector (40–46, 60, 61, 144, 145), and some ‘structural’ work inequalities in Swedish society (such as in leadership roles, gender biased training, work orientations and unpaid home care) (46–51, 55) and thus a significant change seems unlikely. In any case, any new positions for one sex will be at the cost of the other, so no new jobs can be thus created.^2^

There are four other ways through which this plan may increase employment:

1. More generous parental leave regulation by the City (17, p. 18) will create gaps to be filled with part or full-time workers (128, 146)
2. Academic programs and research (17, p. 11, 128, 147) can create jobs and careers.
3. Gender equity has been shown to enhance growth in the long-term (98, 100, 148, 149), which in turn may create jobs and business opportunities.
4. The private sector may follow the city in providing education on gender, more generous parental leaves and better balanced recruitment and employment. Evidence was found of a good corporate predisposition toward gender equity in Sweden and the province of Scania (Skåne) where Malmö is the capital (60, 145, 149, 150), and research on favorable impact on workers′ physical and mental health may further support this trend (57, 151).

However, given the absence of a direct call for job creation, the overall contribution of the plan on employment is very unlikely to exceed 1% of the city′s 201,123 residents (see footnote 1) aged 16–64 (24, 137, 138), of whom over 40% (2010) are not employed (23, 24, 39, 40, 131, 137, 138, 140, 141).^3^ *PEI Score: 1 (1–2)*

Q7. Does the plan promote constituent participation in horizontal and vertical networks? Does the plan create links between the community members/citizens/residents and between them and the authorities of the city?

Promotion of networking of constituents:

  0) No

  1) all for <1%

  2) indirect for 1–10%

  3) indirect for >10%

  4) explicit/direct for 1–10%

  5) explicit/direct for >10%

According to the evidence search:

The plan calls directly for the responsibility of the City to support gender equity contact persons of peripheral administrations and companies through networking. This will happen through personal communication, an existing website (malmo.se/jamstalldhet) and the city intranet (KomIn) (17, p. 13). In this way, the existing horizontal and vertical networks of the City will be enhanced for the coordinators and leaders involved in the project which will probably not account for more than a few hundred constituents (less than 1% of the constituency) (23).

Indirectly, GEIDP aims to put new emphasis on the issue, by spreading knowledge and serving as a model for wider equity in the society. The discussions and seminars are likely to improve the communication between the many groups concerned with gender equity, institutions, agencies and businesses active in Malmö and between those and the City. Around 100 women’s and men′s groups actively pursue gender equity in Scania, with over 10,000 local members (23, 73–75, 77). Although this could include duplicate memberships, the number may easily exceed 1% of the constituency (3,000 members).

Given the large number of existing groups, it is unlikely that many new groups and networks will be formed because of the plan. Therefore, the proportion of constituents that the plan will indirectly motivate to participate in these links is unlikely to exceed 10% of the resident population (30,000 members) (23, Q5). More accurate statistics of residents involved in groups and networks in Malmö would be useful in this regard. *PEI Score: 2 (1–3)*

Q8. Are hard to reach, vulnerable or disadvantaged populations being considered and affirmatively protected and empowered (including vulnerable gender and age groups, socially/physically/economically disadvantaged individuals, groups and communities)?

  0) No

    Yes,

  1) some for <1%

  2) indirect for 1–10%

  3) indirect for >10%

  4) explicit/direct for 1–10%

  5) explicit/direct for >10%

Despite living in one of world’s most equitable societies, many women in Sweden can still be considered disadvantaged and vulnerable. Seventy two percent of all leaders and 82% of board members of publicly listed companies are men. Only 16% of professors are women and a woman’s average salary is 8% lower than a man’s. The aggregate total income of women is 70% of men’s while they contribute three times more in unpaid services (55, 152). One in five women feels she is being treated unequal at work and one in four is unhappy with gender equity in this country (60, 62). There are over 25,000 crime reports, mostly by intimate partners, and around 6,000 rapes against women every year (55).

Malmö is 37th among 290 municipalities on the SCB’s gender equity index (Jämindex), last of the major cities (28). Some recent prominent crimes have raised public attention to violence against women (153–157). Forty percent of its population has an immigrant background and 30% has actually been born abroad (23). Immigrant women often face even more challenges toward employment and empowerment (24).

Some men are also disadvantaged. Only 22% of parental leaves go to men (18, 55), who are also more likely to spend holidays away from family (146). Some feel unhappy with the state’s ‘affirmative action’ for gender equity, and demand same ‘game rules’ instead of equitable ones (63).

The plan calls directly for improvement in the both genders situation among the City’s employees first, and through the handling of public matters to all the gender-disadvantaged residents of Malmö, clearly more than 10% of the population (122, Q5). *PEI Score: 5 (4–5)*

Q9. Does the policy provide for or will there most likely be adequate financial, human and other resources?

  0) no provision

  1) inadequate, central

  2) inadequate, mixed or peripheral

  3) adequate, central

  4) adequate, mixed (central and peripheral)

  5) adequate, peripheral

According to the evidence search:

Central vs. Peripheral: According to the plan, the City Council has the overall responsibility for the funding of the plans goals by the annual budget (17, p. 14). The peripheral administrations and boards are made responsible to allocate budget and other resources for their own gender equity targets and instructions are given for gender budgeting (17, pp. 14, 23) but the funds are provided by the City budget (124, p. 47).

There is no provision for peripheral mobilization of funds for the implementation and follow up of the integration of gender equity at the city departments and agencies. However, the peripheral administrations and companies are asked to assign contact persons responsible for the coordination, and educators will be needed for the necessary training on gender issues (see Q5 and 6). According to the City′s recruitment report, many of these human resources are recruited among local young people and minorities (122, p. 5). No mention is made for any prioritization of members of the recruiting agencies’ communities, where in any case it may be difficult to find the expertise needed for these positions (125).

In conclusion, the plan calls mainly for central financial and human resources.

Sufficient vs. insufficient: Significant funds have already been allocated to advance the plan’s aim for education (25, 125, 128, 130, 147). Malmö City has sufficient financial assets to fund this plan (124, p. 61) and relevant commitments are made in the 2012 budget document (124, p. 8, 24, 37). Given the current economic situation and political will (80–82, 130, 158) in Malmö, funds and fixed assets are thus likely to be sufficient. Given the local and national academic environment′s focus on gender issues (65, 66), knowledge and human resources are also most likely to be available. *PEI Score: 3 (2–4)*

Q10. How will the policy plan be evaluated and adapted?

  0) By some non-legitimate leader/authority, non-transparently

  1) no plan

  2) evaluated and adapted centrally, quantitative methodology

  3) evaluated and adapted centrally, quantitative/qualitative methodology

  4) evaluated and adapted centrally and peripherally, quantitative methodology

  5) evaluated and adapted centrally and peripherally, quantitative/qualitative and/or participatory methodology

According to the evidence search:

The plan calls for annual, gender disaggregated statistics of measurable targets and consequence reports from the city boards and administrations to be continuously created, and an annual revision update report and analysis will be presented by the city board (25, p. 14). The City will be responsible for the overall follow-up of gender equity integration′s progress by the city administrations and also provide support and coordination (25, p. 15). In addition to the statistics, the plan explicitly asks for qualitative equity analyses, in order to sample the ‘residents and users experiences through different kinds of surveys’ (25, p. 24).

In addition, the importance of both quantitative and qualitative aspects are raised in the national policy document on gender equity (16, p. 86) and previous City, State and private evaluations of the gender equity situation have included both qualitative and quantitative methods (159–162), and the same applies for local and national related academic studies (64). Thus, it can be assumed with relative certainty that the plan will be evaluated holistically.

The City Council must approve changes in the plan (25, p. 14). However, as adequate authority is delegated peripherally for implementation, several city departments and administrations have already formulated and adapted the plan to their situations (112–117), so this is also likely to happen after the evaluations. *PEI Score: 5 (4–5)*

Total score: 32 (23–39) or 64% (46–78%). The policy plan can be best described as supportive of empowerment, ranging between the supportive and enabling PEI policy categories.

**Complete Evaluation Report Bibliography**

1. World Health Organization (WHO) (2012). Women and gender equity. Available from: http://www.who.int/social_determinants/themes/womenandgender/en/index.html [cited 5 August 2012].
2. Sen G, Östlin P, George A. Unequal, unfair, ineffective and inefficient – gender inequity in health: why it exists and how we can change it Report of the Women and Gender Equity Knowledge Network of the Commission on Social Determinants of Health; 2007.
3. Fung A, Wright E. Deepening democracy: innovations in empowered participatory governance. Politic Soc 2001; 29: 5–41.
4. Pratchett L, Durose C, Lowndes V, Smith G, Stoker G, Wales C. Empowering communities to influence local decision making: a systematic review of the evidence. London: Department for Communities and Local Government 2009.
5. Kabeer N. Resources, agency, achievements: reflections on the measurement of women’s empowerment. Dev Change 1999; 30: 435–64.
6. Kim J, Paul P, Barnet T, Watts C. Exploring the role of economic empowerment in HIV prevention. AIDS 2008; 22: 57–71.
7. Woodall J, Raine G, South J, Warwick-Booth L. Empowerment and health & well being: evidence review. Leeds: Centre for Health Promotion Research, Leeds Metropolitan University; 2010.
8. Briones-Vozmediano E, Vives-Cases C, Peiro-Perez R. Gender sensitivity in national health plans in Latin America and the European Union. Health Policy 2012; 106: 88–96.
9. Stewart DE, Dorado LM, Diaz-Granados N, Rondon M, Saavedra J, Posada-Villa J, et al. Examining gender equity in health policies in a low- (Peru), middle- (Colombia), and high- (Canada) income country in the Americas. J Public Health Policy 2009; 30: 439–54.
10. Garcia-Calvente MM, Castano-Lopez E, Mateo-Rodriguez I, Maroto-Navarro G, Ruiz-Cantero MT. A tool to analyse gender mainstreaming and care-giving models in support plans for informal care: case studies in Andalusia and the United Kingdom. J Epidemiol Community Health 2007;61(Suppl 2): ii32–38.
11. European Commission (EC) (2007). Report on equality between women and men – 2007. Luxembourg: Office for Official Publications of the European Communities.
12. Council of European Municipalities and Regions and its Partners (2006). The European charter for equality of women and men in local life.
13. United Nations Development Programme (UNDP) (1995). Human Development Report. Available from: http://hdr.undp.org/en/reports/global/hdr1995/chapters/ [cited 8 June 2012].
14. Central Intelligence Agency (CIA) (2012). The World Factbook. Available from: https://www.cia.gov/library/publications/the-world-factbook/rankorder/2172rank.html [cited 8 June 2012].
15. United Nations Development Programme (UNDP) (2011). Human Development Report 2011. Sustainability and equity: a better future for all.
16. Statens Offentliga Utredningar (SOU 2005:66). Makt att forma samhället och sitt eget liv – jämställdhetspolitiken mot nya mål. Slutbetänkande av Jämställdhetspolitiska utredningen. Stockholm; 2005.
17. Malmö Stad (2011). Utvecklingsplan för jämställdhetsintegrering.
18. TNS-SIFO Surveys (2001). Jämställdhet. Available from: http://www.tns-sifo.se/rapporter-undersokningar/senaste-undersokningarna/2001/jaemstaelldhet-/ [cited 5 August 2012].
19. Malmö Stad (2012). Malmöfakta. Available from: http://malmo.se/Kommun--politik/Om-oss/Statistik-om-Malmo.html [cited 7 August 2012].
20. Malmö Stad (2012). Malmöbor med utländsk bakgrund. Available from: http://www.malmo.se/download/18.6e1be7ef13514d6cfcc800030664/Malm%C3%B6bor+med+utl%C3%A4ndsk+bakgrund.pdf [cited 25 July 2012].
21. Gavriilidis G, Östergren P. Evaluating a traditional medicine policy in South Africa: phase 1 development of a policy assessment tool. Glob Health Action 2012; 5: 17271.
22. Stigendal M, Östergren P-O, editors. Malmö’s path towards a sustainable future: health, welfare and justice. Commission for a socially sustainable Malmö. Malmö: Commission for a socially sustainable Malmö; 2013.
23. Malmö Stad (2012). Aktuellt om: Malmös befolkningsutveckling 2011 Stadskontoret.
24. Borg A. Dagen. Sekulär stat inte alltid bäst; 2012. Available from: http://www.dagen.se/nyheter/borg-sekular-stat-inte-alltid-bast/ [cited August 2012].
25. Malmö Stad (2012). Framsteg i arbetet för ett jämställt Malmö. Available from: http://www.malmo.se/Nyheter/Centrala/3-21-2012-Framsteg-i-arbetet-for-ett-jamstallt-Malmo.html [cited 5 August 2012].
26. Sanderson I. Evaluation, policy learning and evidence-based policy making. Publ Admin 2002; 80: 1–22.
27. United Nations Development Programme (UNDP) (2012). Human Development Reports, Indices and Data OECD. Available from: http://hdr.undp.org/en/statistics/indices/gdi_gem/ [cited 7 August 2012].
28. SVT.se (2006). Jämindex – Hela Landet (SvT). Available from: http://www.svt.se/content/1/c6/67/88/72/jamindexhelalandet.pdf [cited 4 August 2012].
29. Organisation for Economic Co-operation and Development (OECD) (2012). Gender indicators: what, why and how?; Available from: http://www.oecd.org/social/genderequalityanddevelopment/43041409.pdf [cited 7 August 2012].
30. Spinakis A, Anastasiou G, Panousis V, Spiliopoulos K, Palaiologou S, Yfantopoulos J. Expert review and proposals for measurement of health inequalities in the European Union – Full Report. European Commission Directorate General for Health and Consumers. Luxembourg; 2011. Available from: http://ec.europa.eu/health/social_determinants/docs/full_quantos_en.pdf [cited 7 August 2012].
31. Stiglitz J. The Stiglitz Report: reforming the international monetary and financial systems in the wake of the global crisis, with members of the commission of experts of the President of the United Nations General Assembly on Reforms of the International Monetary and Financial System; 2012.
32. Landsorganisationen i Sverige (2009). Ny LO-undersökning – kraftigt stöd för jämställda löner. Available from: http://www.lo.se/home/lo/home.nsf/unidView/EF9F61E9223FC5AAC125728A002BDA58 [cited 5 August 2012].
33. Åsblad M. Dagensarena. Svenskar vill ha jämställdhet – men väljer det inte; 2012. Available from: http://www.dagensarena.se/innehall/svenskar-vill-ha-jamstalldhet-men-valjer-det-inte [cited August 2012].
34. Aftonbladet (2010). Bara två procent tycker jämställdhet är viktigt. Available from: http://www.aftonbladet.se/wendela/article12285311.ab [cited 5 August 2012].
35. Tro och politik. Sista ordet: Dags att ta fajten om jämställdheten. October 2011. Available from: http://troochpolitik.se/?p=212031 [cited 5 August 2012].
36. Jakobsson N, Kotsadam A. Do attitudes toward gender equality really differ between Norway and Sweden? J Eur Soc Policy 2010; 20: 142–59.
37. Kvinnojouren (2012). Jämställdhet i Sverige. Available from: http://www.kvinnojouren.se/fakta/feminism-och-jamstalldhet/jamstalldhet-i-sverige [cited 5 August 2012].
38. Adman P. Investigating political equality: the example of gender and political participation in Sweden. Acta Politica 2011; 46.
39. Statistiska Centralbyrån (SCB) (2012). Entreprenörskap och företagande. Available from: http://www.scb.se/Pages/ThematicTables.aspx?id=334633 [cited August 2012].
40. Statistiska Centralbyrån (SCB) (2012). Ekonomisk jämställdhet. Available from: http://www.scb.se/Pages/ThematicTables.aspx?id=334771 [cited 5 August 2012].
41. Carlsson-Kanyama A, Julia I, Rohr U. Unequal representation of women and men in energy company boards and management groups: are there implications for mitigation? Energ Pol 2010; 38: 4737–40.
42. Seierstad C, Healy G. Women’s equality in the Scandinavian academy: a distant dream? Work Employ Soc 2012; 26: 296–313.
43. Hearn J, Nordberg M, Andersen K, Balkmar D, Gotzen L, Klinth R, et al. Hegemonic masculinity and beyond: 40 years of research in Sweden. Men Masc 2012; 15: 31–55.
44. Lindberg L, Riis U, Silander C. Gender equality in Swedish higher education: patterns and shifts. Scand J Educ Res 2011; 55: 165–79.
45. Sainsbury D, Bergqvist C. The promise and pitfalls of gender mainstreaming the Swedish case. Int Fem J Polit 2009; 11: 216–34.
46. Enberg B, Stenlund H, Sundelin G, Ohman A. Work satisfaction, career preferences and unpaid household work among recently graduated health-care professionals – a gender perspective. Scand J Caring Sci 2007; 21: 169–77.
47. Tunberger P, Sigle-Rushton W. Continuity and change in Swedish family policy reforms. J Eur Soc Policy 2011; 21: 225–37.
48. Thomas J, Hildingsson I. Who’s bathing the baby? The division of domestic labour in Sweden. J Fam Stud 2009; 15: 139–52.
49. Thornqvist C. Family-friendly labour market policies and careers in Sweden – and the lack of them. Symposium on Critical Perspectives on Careers and Family-Friendly Policies. Br J Guid Counsell 2006; 34: 309–26.
50. Rothstein B. The reproduction of gender inequality in Sweden: a causal mechanism approach. Gender Work Organ 2012; 19: 324–44.
51. Bernhardt E, Noack T, Lyngstad T. Shared housework in Norway and Sweden: advancing the gender revolution. J Eur Soc Policy 2008; 18: 275–88.
52. Olah L, Bernhardt E. Combining childbearing and gender equality. Demogr Res 2008; 19: 1105–44.
53. Langvasbraten T. A Scandinavian model? Gender equality discourses on multiculturalism. Soc Polit 2008; 15: 32–52.
54. Ahlberg J, Roman C, Duncan S. Actualizing the ‘Democratic family’? Swedish policy rhetoric versus family practices. Soc Polit 2008; 15.
55. Statistiska Centralbyrån (SCB) (2010). Jämställdhetsstatistik. På tal om kvinnor och män. Lathund om jämställdhet.
56. Kotsadam A, Finseraas H. The state intervenes in the battle of the sexes: causal effects of paternity leave. Soc Sci Res 2011; 40: 1611–22.
57. Norström L, Lindberg L, Månsdotter A. Could gender equality in parental leave harm off-springs’ mental health? A registry study of the Swedish parental/child cohort of 1988/89. Int J Equity Health 2012; 11: 11–19.
58. Jonsson P, Schmidt I, Sparring V, Tomson G. Gender equity in health care in Sweden – Minor improvements since the 1990s. Health Policy 2006; 77: 24–36.
59. Haglund B, Köster M, Nilsson T, Rosén M. Inequality in access to coronary revascularization in Sweden. Scand Cardiovasc J 2004; 38: 334–9.
60. Manpower (2011). Undersökning Work Life om jämställdhet i Norge och Sverige: Var fjärde kvinna missnöjd med jämställdheten. Available from: http://www.manpower.se/mpnet3/Content.asp?NodeID=57728&ref=SWEDEN_NORDIC [cited 18 July 2012].
61. Ekonomi Sverige (2011). Hur jämställda är vi? Available from: http://www.ekonomisverige.se/nyheter/hur-jamstallda-ar-vi/ [cited 18 July 2012].
62. Jämställ.nu (2012). Var femte kvinna har någon gång känt sig könsdiskriminerad på arbetsplatsen. Available from: http://www.jamstall.nu/aktuellt_2/nyhetsarkiv/maj/var-femte-kvinna-har-nagon-gang-kant-sig-konsdiskriminerad-pa-arbetsplatsen. [cited 15 July 2012].
63. Pelle Billing. Pelle Billing; 2012. Available from: http://www.pellebilling.se/ [cited 7 July 2012].
64. Uppsatser.se (2012). Sökning: “Undersökning jämställdhet Malmö.” Available from: http://www.uppsatser.se/om/unders%C3%B6kning+av+j%C3%A4mst%C3%A4lldhet+Malm%C3%B6/ [cited 29 July 2012].
65. Lund University (2012). Centre for Gender Studies. Available from: http://www.genus.lu.se/o.o.i.s/4737 [cited August 2012].
66. Malmö University (2012). Research themes. Available from: http://www.mah.se/english/Schools-and-faculties/Faculty-of-Culture-and-Society/DepartmentsSchools/Department-of-Global-Political-Studies/Research/Research-themes/ [cited August 2012].
67. Pew Research Center (2010). Pew global attitudes project. Gender equality Universally Embraced, but inequalities acknowledged. Available from: http://www.pewglobal.org/2010/07/01/gender-equality/ [cited 18 July 2012].
68. Kohei Y. Gender perceptions in Southeast Asian countries: findings from JICA-RI value surveys. World Development Report 2012. Gender Equality and Development, background paper; 2011; 2012.
69. Feministen (2012). Jämställdhetshistoria-Jämo. Available from: http://www.feministen.se/a/jamstalldhetshistoria-jamo [cited 8 August 2012].
70. Sveriges kvinno- och tjejjourers riksförbund (SKR) (2011). Historiska årtal i den svenska jämställdhetens historia. Available from: http://www.kvinnojouren.se/fakta/feminism-och-jamstalldhet/historiska-artal [cited 7 August 2012].
71. United Nations Development Programme Sweden (2012). TEMA: Jämställdhet och kvinnors rättigheter. Available from: http://www.undp.se/kvinnors-rattigheter/ [cited 10 August 2012].
72. Commission of the European Communities (2011). A Roadmap for equality between women and men, 2006–2010.
73. Interenationella Kvinnoföreningen i Malmö (IKF) (2012). Lokalt resurcentrum för kvinnor i Öresundsregion. Available from: http://www.ikf.se/ [cited 7 August 2012].
74. Femcenter (2012). Centrum för kvinnor utsatta för våld. Available from: http://www.femcenter.se/ [cited 5 August 2012].
75. Sveriges Kvinnolobby (2012). Nyheter från European Women’s Lobby. Available from: http://www.sverigeskvinnolobby.se/ [cited 15 August 2012].
76. Samarbeta (2012). Samarbeta. Available from: http://samarbeta.org/ [cited 5 August 2012].
77. Kvinnor nätverk I Skåne (2012). ResursCentrum för kvinnor i Skåne. Available from: http://www.skane.se/upload/Webbplatser/R_Centrum/Dokument/N%C3%A4tverkskatalogn%C3%A4tet30aug05.pdf [cited 7 August 2012].
78. Män för jämställdhet (2012). Malmö. Available from: http://www.mfj.se/malmo [cited 7 August 2012].
79. Ladyfest (2012). Bokare har stort ansvar för jämstäldhet. Available from: http://www.ladyfest.se/malmo/2012/07/13/bokare-har-stort-ansvar-for-jamstalldhet/ [cited 5 August 2012].
80. S-kvinnor i Skånedistriktet (2012). Socialdemokraterna. Available from: http://www.s-info.se/association/default.asp?id=404 [cited 7 August 2012].
81. Nya Moderaterna Malmö (2012). Jämställdhet. Available from: http://xn--malmmoderaterna-ctb.se/jamstalldhet-3/ [cited 7 August 2012].
82. Kristdemokraterna (2012). Jämställdhet. Available from: http://www.kristdemokraterna.se/varpolitik/politikomraden/jamstalldhet [cited 8 August 2012].
83. The Swedish Confederation for Professional Employees (TCO) (2012). Jämställdhet. Available from: http://www.tco.se/Templates/Page2____706.aspx [cited 10 August 2012].
84. Actionaid (2012). Jämställdhet Available from: http://www.actionaid.se/omraden/kvinnor_och_flickor/ [cited 4 August 2012].
85. Sörlin A, Lindholm L, Ng N, Öhman A. Gender equality in couples and self-rated health – a survey study evaluating measurements of gender equality and its impact on health. Int J Equity Health 2011; 10: 37.
86. Westin M, Westerling R. Health and healthcare utilization among single mothers and single fathers in Sweden. Scand J Public Health 2006; 34: 182–9.
87. Galtry J. The impact on breastfeeding of labour market policy and practice in Ireland, Sweden, and the USA. Soc Sci Med 2003; 57: 167–77.
88. Floderus B, Hagman M, Aronsson G, Marklund S, Wikman A. Work status, work hours and health in women with and without children. Occup Environ Med 2009; 66: 704–10.
89. Floderus B, Hagman M, Aronsson G, Gustafsson K, Marklund S, Wikman A. Disability pension among young women in Sweden, with special emphasis on family structure: a dynamic cohort study. BMJ Open 2012; 2.
90. Sörlin A, Ohman A, Lindholm L. Sickness absence in gender-equal companies: a register study at organizational level. BMC Public Health 2011; 11: 548.
91. Östlin P, Eckermann E, Mishra U, Nkowane M. Gender and health promotion: a multisectoral policy approach. Health Promot Int 2006; 21: 25–35.
92. Backhans M, Lundberg M, Månsdotter A. Does increased gender equality lead to a convergence of health outcomes for men and women? A study of Swedish municipalities. Soc Sci Med 2007; 64: 1892–903.
93. Lister R. A Nordic Nirvana? Gender, citizenship, and cocial justice in the Nordic Welfare States. Social politics. Conference of the Nordic-Council-of-Ministers. May, 2006. Oslo, Norway. Nordic Council Ministers. Soc Polit 2009; 16: 242–78.
94. Forbes J, Ohrn E, Weiner G. Slippage and/or symbolism: gender, policy and educational governance in Scotland and Sweden. Gender Educ 2011; 23: 761–76.
95. Barry J, Berg E, Chandler J. Movement and coalition in contention: gender, management and academe in England and Sweden. Gender Work Organ 2012; 19: 52–70.
96. Gornick J, Meyers M. Creating gender egalitarian societies: an agenda for reform. Polit Soc 2008; 36: 313–49.
97. Tomlinson J. Gender equality and the state: a review of objectives, policies and progress in the European Union. Int J Hum Resour Manag 2011; 22: 3755–74.
98. The World Bank. World Development Report: the political economy of gender reform. World Dev Rep 2012; 2012: 330–58.
99. The World Bank News and Broadcast (2012). Setting international standards for gender equality in the private sector. The Gender Equity Model. Available from: http://web.worldbank.org/WBSITE/EXTERNAL/NEWS/0,,contentMDK:23203941~pagePK:34370~piPK:34424~theSitePK:4607,00.html [cited 5 August 2012].
100. OECD (2012). Bridge development-gender. Available from: http://www.oecd.org/dataoecd/46/47/43041409.pdf [cited 7 August 2012].
101. Olah L. Should governments in Europe be more aggressive in pushing for gender equality to raise fertility? The second ‘YES’. Demogr Res 2011; 24: 179–200.
102. Bernardi L, Hetze P. Preface to the Rostock debate on demographic change. Demogr Res 2011; 24: 175–8.
103. Neyer G. Should governments in Europe be more aggressive in pushing for gender equality to raise fertility? The second ‘NO’. Demogr Res 2011; 24: 225–50.
104. Ronsen M, Skrede K. Can public policies sustain fertility in the Nordic countries? Lessons from the past and questions for the future. Demogr Res 2010; 22: 321–46.
105. Malmö stad (2012). Så arbetar vi med Jämställdhetsintegrering. Available from: http://www.malmo.se/Kommun--politik/Sa-arbetar-vi-med.../Jamstalldhetsintegrering/Bakgrund.html [cited 10 August 2012].
106. Malmö Stad (2010). Rapport – om nuläget i Malmö stad inför framtagande av förslag till utvecklingsplan för jämställdhetsintegrering.
107. Malmö stad S (2011). Tjänsteutlåtande KS-KOM-2010-00632: Utvecklingsplan för jämställdhetsintegrering i Malmö stad 2011–2020.
108. Malmö Stad (2001). Övergripande plan för jämställdhetsarbetet i Malmö stad.
109. Regeringen (2011). Integration och jämställdhet.
110. Jämställdhetsombudsmannen (JämO) (2007). Grundbok – att arbeta fram en jämställdhetsplan/JämO.
111. Glans H, Rother B. Dragkraft! Om jämställdhetsintegrering och ESF Jämt; 2011.
112. Malmö Stad (2012). Jämställdhet och mångfaldsplan; Limhamn-Bunkeflo stadsdelsförvaltning.
113. Malmö Stad (2012). Jämställdhet och mångfaldsplan: Oxie Stadsdelsförvaltning. 2012–2014.
114. Malmö Stad (2010). Slutrapport JÄMSTÄLLDHET – En ny ambition Rosengård stadsdelsförvaltning.
115. Lund University (2009). Handlingsplan för jämställdhet, likabehandling och breddad rekrytering vid Teaterhögskolan i Malmö.
116. Nordregio (2009). Att stärka tryggheten i stads- och tätortsmiljöer ur ett jämställdhetsperspektiv.
117. Malmö Stads Gatukontoret (2010). Hållbar jämställdhet inom Framtidens kollektivtrafik.
118. Länsstyrelsen i Skåne Län (2012). Trygghet och jämställdhet i samhällsplaneringen. Available from: http://www.lansstyrelsen.se/skane/Sv/samhallsplanering-och-kulturmiljo/planfragor/aktuella-planeringsfragor/tryggt-och-jamnt/Pages/tryggt-och-jamnt.aspx
119. Job och uddannelse i Öresundsregionen (2011). Öresundsregionen: tillgänglighet och mobilitet av arbetskraften. Available from: http://jobuddannelse.net/files/dokumenter/artikler/oresundsregionen2c20tillganglighet20och20mobilietet20av20arbetskraften_0.pdf [cited 5 August 2012].
120. 120.Statistiska Centralbyrån (SCB) (2012). Arbetsmarknad, Befolkningen 16+ år efter kommun, sysselsättning (förvärvsarbetande), ålder och kön. Available from: http://www.ssd.scb.se/databaser/makro/visavar.asp?yp=duwird&xu=c5587001&lang=1&langdb=1&Fromwhere=S&omradekod=AM&huvudtabell=YREG1&innehall=Befolkningen&prodid=AM0208&deltabell=K1&fromSok=&preskat=O [cited 5 August 2012].
121. Malmö Stad (2011). Personal facta. Available from: http://www.malmo.se/Medborgare/Jobb--praktik/Jobba-hos-oss/Personalfakta.html [cited 5 August 2012].
122. Malmö Stad (2011). Malmö stads personalredovisning. Available from: http://redovisningar.malmo.se/redovisning/assets/personalredovisning.pdf [cited 5 August 2012].
123. Malmö Stad (2011). Utbildningsförvaltningen. Jämställdhetsplan.
124. Malmö Stad (2012). Budget 2012. Kommunfullmäktiges Bihang Nr 801/2012.
125. Malmö Stad (2011). Lediga-jobb. Utbildare/genuspedagog i genus- och jämställdhet till FoU Malmö-utbildning. Available from: http://webapps03.malmo.se/lediga-jobb/item/7505F3E0-DB22-42AB-B556-71404CC576B6 [cited 5 August 2012].
126. Statistiska Centralbyrån (SCB) (2011). Befolkningens utbildning. Utbildning för befolkningen efter hemkommun. Available from: http://www.scb.se/Pages/ProductTables____9575.aspx [cited 5 August 2012].
127. Malmö Stad (2010). Stort intresse för jämställdhet bland Malmös skolor och förskolor. 2010. Available from: http://www.malmo.se/Medborgare/Forskola—utbildning/Nyhetsarkiv-Forskola—utbildning/Arkiv/1-20-2010-Stort-intresse-for-jamstalldhet-bland-Malmos-skolor-och-forskolor-.html [cited 5 August 2012].
128. Malmö högskola (2009). Nästan 1,5 miljoner för att studera jämställdhet. Available from: http://www.mah.se/Nyheter/Nyheter/Nastan-15-miljoner-till-jamstalldhet/ [cited 5 August 2012].
129. Malmö högskola (2010). Delegationen för jämställdhet gästade Malmö högskola. Available from: http://www.mah.se/Nyheter/Nyheter-2010/Delegationen-for-jamstaldhet-gastade-Malmo-hogskola/ [cited 5 August 2012].
130. Skanskan.se (2012). Jämställdhet rörde upp politiska känslor; 2012. Available from: http://www.skanskan.se/article/20120412/MALMO/704129723/-/jamstalldhet-rorde-upp-politiska-kanslor [cited 5 August 2012].
131. Malmö högskola (2012). Chefskap – jämställdhet. Available from: http://mah.se/cta/ikea/chefskap_jamstalldhet [cited 5 August 2012].
132. Gottzén L, Jonsson R. Andra män (Ny). Maskulinitet, normskapande och jämställdhet. Gleerups; 2012. Available from: http://webbshop.gleerups.se/se/uni-_hog/socialt_arbete-_vardutbildningar/sociologi/andra_man.ecp?groupdetail=true&PositionId=15&product_category_id=1617 [cited 5 August 2012].
133. Malmövänstern v-blog (2012). Malmös läromedel håller inte måttet. Available from: http://malmovanstern.v-blog.se/2012/04/03/malmos-laromedel-haller-inte-mattet/ [cited 8 August 2012].
134. Sydsvenskan (2012). Utbildningar ska skydda kvinnor. Available from: http://www.sydsvenskan.se/malmo/utbildningar-ska-skydda-kvinnor/ [cited 5 August 2012].
135. Langréus A, Nilsson H. Flickor är pojkar är flickor är...- en undersökning om jämställdhet i förskola och skola; 2006.
136. Statistiska Centralbyrån (SCB) (2010). Befolkningens studiedeltagande. Studiedeltagande för befolkningen efter hemkommun. Available from: http://www.scb.se/Pages/ProductTables____9611.aspx [cited 4 August 2012].
137. Malmö Stad (2011). Folkmängd i Malmö efter kön och ålder. Available from: http://www.malmo.se/download/18.72bfc4c412fc1476e02800046044/Folkm%C3%A4ngd+i+Malm%C3%B6+efter+k%C3%B6n+och+%C3%A5lder%2C+1+jan+2011.pdf [cited 5 August 2012].
138. Statistiska Centralbyrån (SCB) (2011). Befolkning. Available from: http://www.scb.se/statistik/_publikationer/OV0904_2011A01_BR_05_A01BR1101.pdf [cited 5 August 2012].
139. Statistiska Centralbyrån (SCB) (2011). Befolkningens utbildning. Utbildningsnivå för befolkningen efter inrikes/utrikes född, kön och åldersgrupp. Available from: http://www.scb.se/Pages/ProductTables____9575.aspx [cited 5 August 2012].
140. JobbMalmö (2012). Resursenheten. Unga kvinnor. Available from: http://www.malmo.se/Medborgare/Jobb—praktik/JobbMalmo/Resursenheten/Unga-kvinnor.html [cited 5 August 2012].
141. Ledarna Sveriges chefsorganisation (2011). Agenda Jämställdhet: ‘Det börjar med chefen’.
142. JobbMalmö (2012). Resursenheten. Krami för kvinnor. Available from: http://www.malmo.se/Medborgare/Jobb—praktik/JobbMalmo/Resursenheten/Krami-for-kvinnor.html [cited 5 August 2012].
143. Bettio F, Verashchagina A. Gender segregation in the labour market: root causes, implications and policy responses in the EU; 2008; B14033.
144. Foretagarna. Jämställt Företagarindex 2012: VAr är företagandet mest (och minst) jämställt?; 2012.
145. Veckans affärer (2012). Bästa företagen på jämställdhet. Available from: http://www.va.se/ledarskap/sveriges-basta-arbetsplatser/basta-foretagen-pa-jamstalldhet-323065 [cited 5 August 2012].
146. Bloggportalen (2011). Jämställdhet nästa undersökning.
147. Malmö högskola (2009). Pengar för jämställdhet finns att söka. Available from: http://mah.se/Nyheter/Nyheter/Jamstalldhet-i-hogskolan/ [cited 5 August 2012].
148. Nilson M, Andersson B. Jämställdhet I Näringslivet. Samlade Erfarenheter Från Tolv Företag i Skåne; 2007.
149. Fiberoptic Valley (2009). Best practise: innovation och jämställdhet Malmö. Available from: http://fiberopticvalley.com/blog/nyheter/best-practise-innovation-och-jamstalldhet-malmo-9-juni/ [cited 5 August 2012].
150. Hemström O. Health inequalities by wage income in Sweden: the role of work environment. Soc Sci Med 2005; 61: 637–47.
151. Krantz G, Lundberg U. Workload, work stress, and sickness absence in Swedish male and female white-collar employees. Scand J Public Health 2006; 34: 238–46.
152. Region Skåne (2010). Regionalt Resurs Centrum för kvinnor. Available from: http://www.skane.se/sv/Webbplatser/Resurscentrum/Jamstalldhet/ [cited 5 August 2012].
153. Kvinnojouren (2012). Kriscentrum för kvinnor i Malmö. Available from: http://www.kvinnojouren.se/jour/kriscentrum-kvinnor-i-malmo [cited 5 August 2012].
154. Sydsvenskan (2012). Karin hjälper utsatta kvinnor. Available from: http://www.sydsvenskan.se/malmo/karin-hjalper-utsatta-kvinnor [cited 20 July 2012].
155. Svenska Dagbladet (2012). Kvinna knivskars till döds i Malmö. Available from: http://www.svd.se/nyheter/inrikes/kvinna-knivskars-till-dods-i-malmo_6861633.svd [cited 20 July 2012].
156. Sydsvenskan (2012). Kvinna misstänkt mördad. Available from: http://www.sydsvenskan.se/malmo/kvinna-misstankt-mordad [cited July 2012].
157. Sveriges Radio Malmö (2012). Misshandlad kvinna avled.
158. Malmovänstern (2012). Jämställdhet. Available from: http://malmovanstern.v-blog.se/category/jamstalldhet/ [cited 8 August 2012].
159. Malmö Stads jämställdhetspolitiken (2003). Utvärderingsrapport om förstudie till projektet Jämställdhet. Malmö.
160. Sveriges Kommuner och Landsting (2011). Program för Hållbar Jämställdhet, Resultatrapport För Perioden 2008–2010. Stockholm.
161. Riksidrottsförbundet (2011). Utvärdering av Riksidrottsförbundets jämställdhetsarbete mellan åren 2005–2010. Stockholm.
162. E(uro)QUALITY Jämställdhetskonsult (2011). Medarbetarenkät; jämställdhet och mångfald. Available from: www.euroquality.se, 2012.

## Appendix 2 Policy Empowerment Index Footnotes

The PEI was previously developed to assess policy elements that have a potential impact on empowerment. It applies logical discussions but, due to lack of evidence on empowering factors’ impacts, not evidence-weighted scores (from 0=minimum to 5=maximum) of empowerment potential (21) and confidence intervals (0–5). In order to make the results more visible) the PEI evaluators attach scores to logical statements. For example, <10% describes ‘a small minority, 10–50% ‘a significant minority’, 50–75% ‘a majority’, 75–90% ‘a big majority’ and 90–100% ‘a vast majority/ almost all’ as previously described (21).

Unless otherwise specified, the score of 0 should be applied when the policy does not benefit the constituents or has a negative impact.

In the case of insufficient information or non-applicable criteria the relevant questions should be removed and the score re-adjusted.

The final score is presented as a proportion of the maximum points (50).

According to the index, a policy can be classified as:

1. Dictative: In this policy type, the policy-makers are addressing a problem that a minority of their constituents know or are concerned about. Mainly expert opinion and central interests guide the policy agenda setting and planning. The plans call for top–down implementations, without significant adaptation to peripheral particularities, needs and participation. The policy does little to build community capacity through education, training or employment opportunities or to protect vulnerable populations. Human, financial and other resources are centrally managed and inadequate. No formative or summative impact evaluations are considered necessary and central, inflexible mechanisms are or are put in place for policy modifications. The policy is interdependent with other dictative policies. PEI: 0–20%

2. Directive: A specific, detailed policy is designed, based on expert opinion and firm strategic goals, after considering public opinion and possible opposition, but mainly based on partisan views (e.g. the ruling party political ideology). Central and peripheral, expert panel committees may contribute to the policy plan. The policy-experts provide a detailed guidance to reach the policy objectives, allowing little deviation in planning and implementation, however taking into consideration local particularities, input and voices of objection, also allowing for some peripheral participation, variation and adaptation to an extent such that the end outputs remain within the prescribed margins. Contrary to the previous level, peripheral authorities are respected although relatively passive partners in the policy planning and implementation, under the firm guidance of the higher legislative framework. The policy builds peripheral capacity to some extent through education, training and provisions for limited employment opportunities and participation in peripheral networks. It protects and empowers affirmatively a minority of vulnerable constituents. Adequate, mainly central resources are planned. The policy is evaluated centrally for predefined outputs and outcomes, using quantitative measurements and can be adapted by central or peripheral inputs, albeit rather inflexibly. It relates and interacts with directive policies. PEI: 21–40%

3. Supportive: The policy addresses a problem that the majority of its constituents know of and are concerned about. Lay representatives together with experts contribute in setting the policy agenda and in policy planning, albeit centrally. A significant part of the implementation depends on peripheral mechanisms and peripheral capacity building is provided. Also, the policy is facilitating wider employment and peripheral network formation and action through legislational provisions and affirmatively supports the majority of vulnerable constituents. The central authority is providing adequate funds, advice, and actively supports peripheral implementation, for example, by providing expert advisers, information and a permissive national policy framework. It evaluates the policy implementation and impact formatively using both quantitative and qualitative methods and flexible, central mechanisms for adaptation exist or are set. The policy relates mainly with other supportive policies. PEI: 41–60%

4. Enabling: An enabling policy frequently addresses a problem that the vast majority of those affected know of and are concerned with. The agenda is set by an interaction of central and peripheral actors, and the policy planning occurs at close collaboration between the center and the periphery. Implementation is mainly delegated to the periphery. The policy also encourages peripheral sustained participation in assessment of needs and relevant action through sustained training and education of constituents, and through promotion of peripheral entrepreneurship, networking and cooperation at and between different levels. Adequate financial, informational and legislative support are combined with local resources and used as catalysts for peripheral involvement, participation and leadership, thereby promoting peripheral capacity development, proactively supporting and empowering the vast majority of vulnerable populations. Peripheral formative evaluation occurs, mainly through quantitative data collection and the policy can be adapted at least at the peripheral level, flexibly. The policy is part of a wider enabling policy matrix. PEI: 61–80%

5. Empowering: The policy is inspired as well as planned and implemented by the constituents under an empowering central and peripheral legislative framework, in a representative, consensual, polyphonic, equitably participatory manner, addressing issues that nearly all the affected constituents are informed and concerned with. It promotes further peripheral achievement and a sustainable, social, democratic development through essential, broad capacity development, through knowledge generation and communication, innovative and expansive peripheral entrepreneurship and asset generation, and through broad, inclusive and active, vertical and horizontal networks/links. Most vulnerable groups are affirmatively empowered. Peripheral participatory needs, formative and summative evaluations are conducted in a holistic manner (quantitative and qualitative, participatory action research) and a continuous feedback drives adaptations flexibly at the peripheral and central-framework level. Related policies follow the same principles. PEI: 81–100% (21).
